# The Implementation in Context (ICON) Framework: A meta-framework of context domains, attributes and features in healthcare

**DOI:** 10.1186/s12961-023-01028-z

**Published:** 2023-08-07

**Authors:** Janet E. Squires, Ian D. Graham, Wilmer J. Santos, Alison M. Hutchinson, The ICON Team, Chantal Backman, Anna Bergström, Jamie Brehaut, Melissa Brouwers, Christopher Burton, Ligyana Korki de Candido, Christine Cassidy, Cheyne Chalmers, Anna Chapman, Heather Colquhoun, Janet Curran, Melissa Demery Varin, Paula Doering, Annette Elliott Rose, Lee Fairclough, Jillian Francis, Christina Godfrey, Megan Greenough, Jeremy M. Grimshaw, Doris Grinspun, Gillian Harvey, Michael Hillmer, Noah Ivers, John Lavis, Shelly-Anne Li, Susan Michie, Wayne Miller, Thomas Noseworthy, Tamara Rader, Mark Robson, Jo Rycroft-Malone, Dawn Stacey, Sharon Straus, Andrea C. Tricco, Lars Wallin, Vanessa Watkins

**Affiliations:** 1 School of Nursing, University of Ottawa, Ottawa, Canada; 2 Clinical Epidemiology Program, Ottawa Hospital Research Institute, Ottawa, Canada; 3 School of Epidemiology and Public Health, University of Ottawa, Ottawa, Canada; 4 School of Nursing and Midwifery, Deakin University, Geelong, Australia; 5 Barwon Health, Geelong, Australia

**Keywords:** Context, Framework, Healthcare, Implementation, Knowledge translation

## Abstract

**Background:**

There is growing evidence that context mediates the effects of implementation interventions intended to increase healthcare professionals’ use of research evidence in clinical practice. However, conceptual clarity about what comprises context is elusive. The purpose of this study was to advance conceptual clarity on context by developing the Implementation in Context Framework, a meta-framework of the context domains, attributes and features that can facilitate or hinder healthcare professionals’ use of research evidence and the effectiveness of implementation interventions in clinical practice.

**Methods:**

We conducted a meta-synthesis of data from three interrelated studies: (1) a concept analysis of published literature on context (*n* = 70 studies), (2) a secondary analysis of healthcare professional interviews (*n* = 145) examining context across 11 unique studies and (3) a descriptive qualitative study comprised of interviews with heath system stakeholders (*n* = 39) in four countries to elicit their tacit knowledge on the attributes and features of context. A rigorous protocol was followed for the meta-synthesis, resulting in development of the Implementation in Context Framework. Following this meta-synthesis, the framework was further refined through feedback from experts in context and implementation science.

**Results:**

In the Implementation in Context Framework, context is conceptualized in three levels: micro (individual), meso (organizational), and macro (external). The three levels are composed of six contextual domains: (1) actors (micro), (2) organizational climate and structures (meso), (3) organizational social behaviour (meso), (4) organizational response to change (meso), (5) organizational processes (meso) and (6) external influences (macro). These six domains contain 22 core attributes of context and 108 features that illustrate these attributes.

**Conclusions:**

The Implementation in Context Framework is the only meta-framework of context available to guide implementation efforts of healthcare professionals. It provides a comprehensive and critically needed understanding of the context domains, attributes and features relevant to healthcare professionals’ use of research evidence in clinical practice. The Implementation in Context Framework can inform implementation intervention design and delivery to better interpret the effects of implementation interventions, and pragmatically guide implementation efforts that enhance evidence uptake and sustainability by healthcare professionals.

**Supplementary Information:**

The online version contains supplementary material available at 10.1186/s12961-023-01028-z.

## Background

Context is critical to successful implementation, as observed in many studies where the success of implementation efforts varied by context. For example, Hogg et al. [1, 2], in trials on the effects of practice facilitation on preventive care delivery, found benefits in capitation-funded practices but not fee-for-service practices. Similarly, Shojania et al. [3] found that point-of-care reminders that targeted inpatient settings resulted in larger improvements in processes of care compared with outpatient settings. More recently, Joseph-Williams and colleagues [4] conducted a realist review (*n* = 29 articles) of scenarios where the characteristics of healthcare professionals, patients, and management, and the broader organizational context can hinder or facilitate implementation of patient decision aids. In 8 [5–12] of the 29 included articles, investigators found that role clarity and appropriate distribution of tasks amongst a multidisciplinary team helped build team cohesion, which then influenced the success of implementing patient decision aids. Similarly, Indraratna and colleagues [13] found, in a process evaluation of the implementation of a new model of care, that lower uptake was attributed to time constraints. Additionally, the effectiveness of implementation efforts is frequently reported to depend on so-called contextual factors that: (i) cause and sustain the problem the intervention is designed to overcome, (ii) influence the susceptibility of the problem to the intervention and (iii) determine how the intervention can work [14]. Despite recognition and evidence that context can mediate the effects of implementation interventions, there is little consistency in the literature concerning what comprises context.

In the field of implementation science, multiple and vastly different definitions of context exist. For example, Øvretveit [15] defines context as all factors that are not part of an intervention. Similarly, Rycroft-Malone et al. [16] define context as “the environment or setting in which the proposed change is to be implemented” (p. 299). Other authors have adopted more specific definitions. For instance, May and colleagues [14] describe context as “the physical, organizational, institutional, and legislative structures that enable and constrain, and resource and realize, people and procedures” (p. 3). Comparably, Pfadenhauer [17] defines context as a set of characteristics and circumstances that consist of active and unique components surrounding an implementation project. A recent scoping review [18] of determinant frameworks used in implementation science found that most (*n* = 15 of 17, 88%) frameworks identified did not provide specific definitions of context. Instead, they defined the concept indirectly by identifying the contextual determinants that may comprise context. Due to this conceptual confusion regarding what constitutes context, researchers and implementation teams continue to investigate and operationalize context differently, seriously hampering progress in implementation science.

There are no implementation frameworks dedicated solely to context. However, several implementation frameworks include context [19–26]. In each of these frameworks, context is considered important to implementation success and is characterized as a multi-dimensional concept. While each of these frameworks provides an important starting point for understanding context within implementation, they offer limited detail, and thus guidance, for identification of the core attributes and features of context that are important to assess and promote implementation success. This study aimed to close this gap by advancing conceptual clarity on context through development of the Implementation in Context (ICON) Framework. ICON is a meta-framework of the context domains, attributes and features that can facilitate or hinder healthcare professionals’ use of research evidence and the effectiveness of implementation interventions in clinical practice. In this paper, we describe ICON and its development.

## Methods

**Fig. 1:**
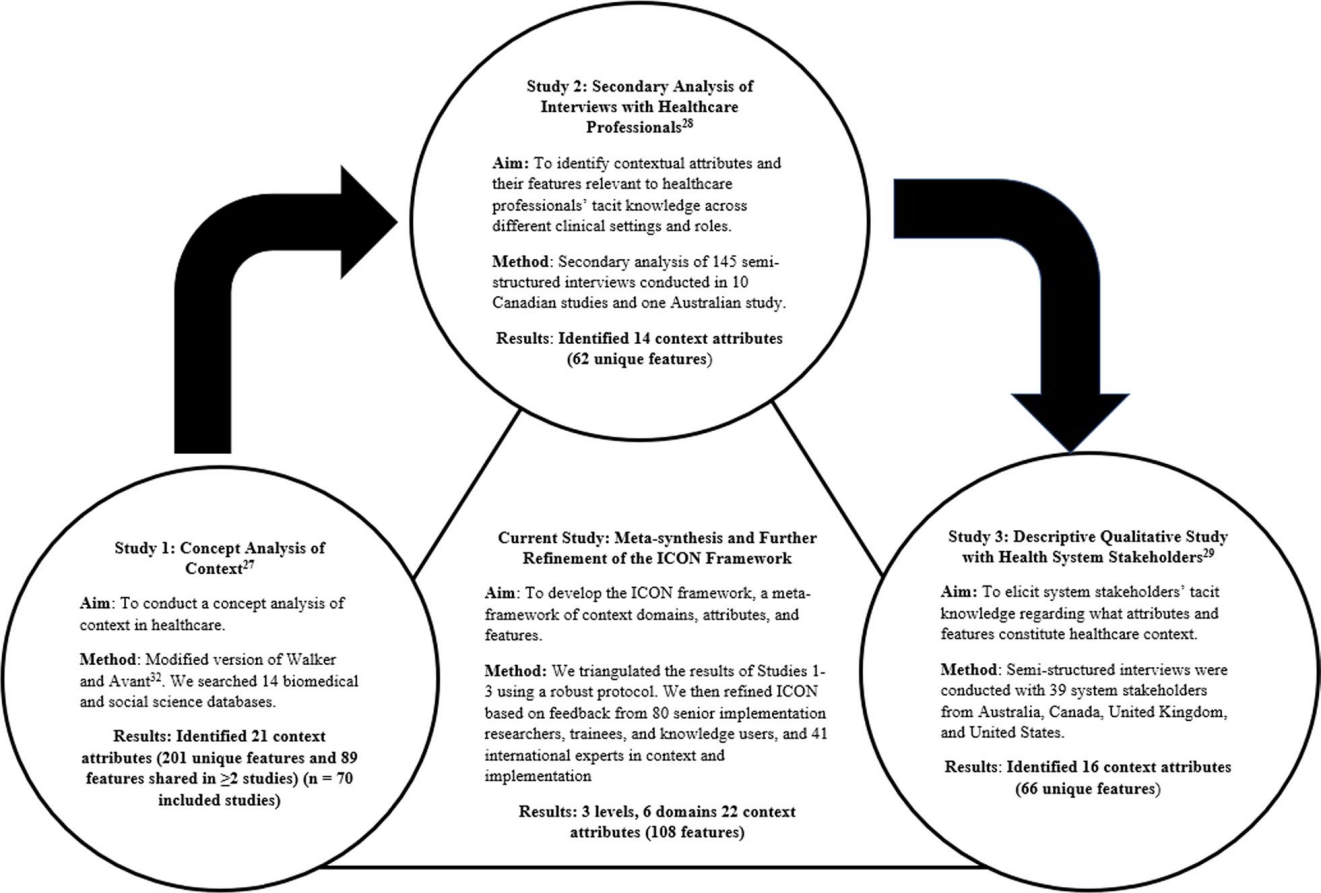
Process for developing the ICON Framework

Figure 1 depicts a summary of the process used to develop ICON. We developed ICON through a meta-synthesis of findings from three interrelated studies we conducted to promote conceptual clarity and understanding of context in implementation. The three studies comprised: (1) a concept analysis of published literature on context [27], (2) a secondary analysis of healthcare professional interviews examining elements of context relevant to their use of research evidence across different settings and clinical behaviours [28] and (3) a descriptive qualitative study with health system stakeholders (change agents/implementation specialists and implementation researchers) to elicit their tacit knowledge of elements of context important to implementation in healthcare [29]. Following the meta-synthesis, ICON was developed and then further refined based on feedback from context and implementation experts. Studies 1–3, which were the building blocks for developing ICON are summarized next; greater detail on the individual methodologies used and the findings of each of the studies are reported in previous publications [27–31]. We used the Enhancing Transparency in Reporting the Synthesis of Qualitative Research (ENTREQ) statement to enhance the accuracy and transparency of reporting our methods (Additional file 1).

### Study 1: Concept analysis of context

In Study 1, we used a modified version of Walker and Avant’s [32] method to conduct a concept analysis of context; our protocol was published [30]. Our modified approach comprised five steps: concept selection, determination of aims, identification of uses of context, determination of its defining attributes and definition of its empirical referents. We systematically searched both biomedical and social science databases from inception to August 2014. These databases covered a wide range of disciplines including health, psychology, social sciences, business and engineering (Additional file 2). Empirical articles were included if a definition and/or attributes or features of context were reported. Theoretical articles were included if they reported a model, theory or framework of context or where context was a component of a model, theory or framework. Double independent screening and data extraction were conducted. Analysis was iterative, involving organizing and reorganizing until an initial framework of domains, attributes and features of context emerged. Our search identified 15 972 citations, of which 70 articles were included in the final analysis. In total, 201 unique context features were identified, of which 89 were shared (reported in two or more studies). The 89 shared features were grouped into 21 defining attributes of context [27].

### Study 2: Secondary analysis of interviews with healthcare professionals

In Study 2, we conducted a secondary analysis of 11 studies on the determinants of using research evidence in clinical practice [28]. The 11 studies comprised 145 interviews conducted from 2007 to 2014 with physicians and nurses from two countries (Canada and Australia) across multiple healthcare settings, which included primary care and different hospital settings (for example, medical and surgical wards, preassessment units, emergency room settings (adult and pediatric), birthing units and intensive care units). Implementation and de-implementation of 11 behavioural practices were also included: hand hygiene, pre-operative assessment, computerized tomography head rules (adult and pediatric), organ donation after cardio-circulatory death, fetal monitoring, red blood cell transfusions, bone mineral density screening, smoking cessation and preconception care. The original interviews were conducted using interview guides informed by the Theoretical Domains Framework [33]. The interview questions were broad, enabling interviewees to spontaneously identify elements of context relevant to their use of evidence in clinical practice. We analysed these data inductively, using constant comparative analysis, to identify attributes of context and their features. Data analysis occurred in three steps. We identified 62 unique features that were grouped into 14 attributes of context. There was considerable consistency in the context attributes identified across the different professional roles and settings [28].

### Study 3: Descriptive qualitative study with health system stakeholders

In Study 3, we conducted a descriptive qualitative study [29]. We undertook semi-structured interviews with health system stakeholders to elicit their tacit knowledge of the attributes and features of context important for improved research use by healthcare professionals. We conducted 39 interviews: 19 interviews with organizational change agents/implementation specialists and 20 interviews with senior researchers in the field of implementation science. Interviews were conducted across four countries: Australia (*n* = 12), Canada (*n* = 14), the United States of America (*n* = 7), and the United Kingdom (*n* = 6). We analysed the data inductively, using thematic content analysis, in four steps: (1) selection of utterances of context, (2) merging of similar codes into features of context, (3) categorizing features of context into higher-level attributes of context and (4) comparison of attributes and their features by country, years of interviewee experience in implementation, and interviewee primary role (organizational change agent or researcher). We identified 66 unique features, which were grouped into 16 attributes of context. Similar to Study 2, there was considerable consistency in the context attributes identified across the four countries and interviewee experience and role [29].

### Meta-synthesis protocol – the current study

To create an initial version of the ICON Framework we conducted a meta-synthesis of the findings from Studies 1–3. We followed the triangulation protocol of Farmer and colleagues [34], designed to synthesize multiple qualitative data sources. This protocol comprises six steps: (1) sorting, (2) convergence coding, (3) convergence assessment, (4) completeness assessment, (5) team member comparison and (6) feedback. Two team members independently undertook all steps of the synthesis and then compared their results, seeking feedback from the larger team during each step. Synthesis occurred at the lowest level of data, features of context, aggregated to the higher levels found within ICON: first attributes of context, then context domains and, finally, levels (micro, meso and macro) of context.

#### Step 1: Sorting

Initially, three separate Microsoft Excel files were created from the findings of Studies 1–3; each file contained a listing of the features produced from the respective study. All three files were reviewed by a data synthesis team consisting of two research assistants and three senior investigators. After sorting and identifying features within the three phases, we compared the features included in Study 2 (healthcare professional interviews) and Study 3 (stakeholder interviews) because they used the same initial coding scheme. Features that were duplicated were merged, resulting in one list of features for Study 2 and Study 3. We then compared this merged list of features to those from Study 1 (concept analysis); duplicate features were again merged. During merging, we retained the definition for the feature that had greater clarity, or in select cases, we merged definitions from the different studies to create a more refined definition.

#### Step 2: Convergence coding

We created a convergence-coding matrix to compare the previously sorted and merged files described above (concept analysis file and the merged healthcare professional and stakeholder interviews file) with respect to the meaning (our definition of the feature) and frequency of the context feature. If a feature was identified in both lists and had similar frequency across them (within a 30% range, as suggested by Farmer and colleagues [34]), this was classified as “full agreement”. If a feature was identified in both lists, but with dissimilar frequencies, this was termed “partial agreement”. If a feature was only identified in one of our lists, this was termed “silence”. The type of convergence was classified as either “full agreement” (defined as full agreement between the sets of results on both elements of comparison, for example, the meaning and frequency are the same), “partial agreement” (defined as agreement on one but not both components, for example, meaning or frequency is the same), or “silence” (defined as one set of results covering the feature while another set of results was silent on the feature) [34].

#### Step 3: Convergence assessment and Step 4: Completeness comparison

Steps 3 and 4 were conducted iteratively. We conducted a convergence assessment by assessing the percent of features with full agreement, partial agreement and silence (as defined above) to gain a global understanding of convergence across the three studies. All discrepancies in convergence were resolved through consensus and in consultation with the investigator team. We recorded the final convergence assessment but kept track of disagreements that occurred. We then examined the final list of features to identify any similar and unique contributions from the three different studies.

#### Step 5: Team member comparison and Step 6: Feedback

Steps 5 and 6 were also conducted iteratively. Throughout the coding, weekly consensus meetings were held to resolve conflicts in consultation with a subgroup of senior investigators. The final list of features was grouped into attributes and further grouped into domains, which were reviewed by the wider interdisciplinary and international ICON investigator team. Refinements to the analysis were made based on team feedback to form the first iteration of the ICON Framework.

### Further refinement of ICON

We sought expert feedback to further refine our initial version of ICON. First, we pursued broad external feedback; we presented the ICON Framework to 80 senior implementation researchers, trainees (PhD students and post-doctoral fellows in implementation) and knowledge users (health system stakeholders, including administrators and representatives of health research funding agencies) from 9 countries (Canada, the United States, Sweden, England, Australia, Saudi Arabia, Norway, the Netherlands, Denmark and China) attending the *2019 Knowledge Utilization Colloquium* [35]. For each defining context attribute in ICON, attendees were asked to consider “When designing an implementation intervention, how important is it to assess this attribute of context?”. Findings from this assessment are in Additional file 3. Second, we sought expert feedback from our large interdisciplinary team. We sent the ICON Framework to the investigators from Studies 1–3 and the meta-synthesis, each of whom are international experts in the fields of context and/or implementation. This comprised sending ICON to a total of 41 people from six countries (Australia, Brazil, Canada, the United Kingdom, the United States and Sweden) and eight disciplinary backgrounds, including clinical epidemiology, dietetics, health and psychological sciences, health leadership, library science, nursing, medicine and rehabilitation. Based on the feedback received, we refined the ICON Framework to produce the current version, presented next.

## Results

### Overview of ICON

**Fig. 2:**
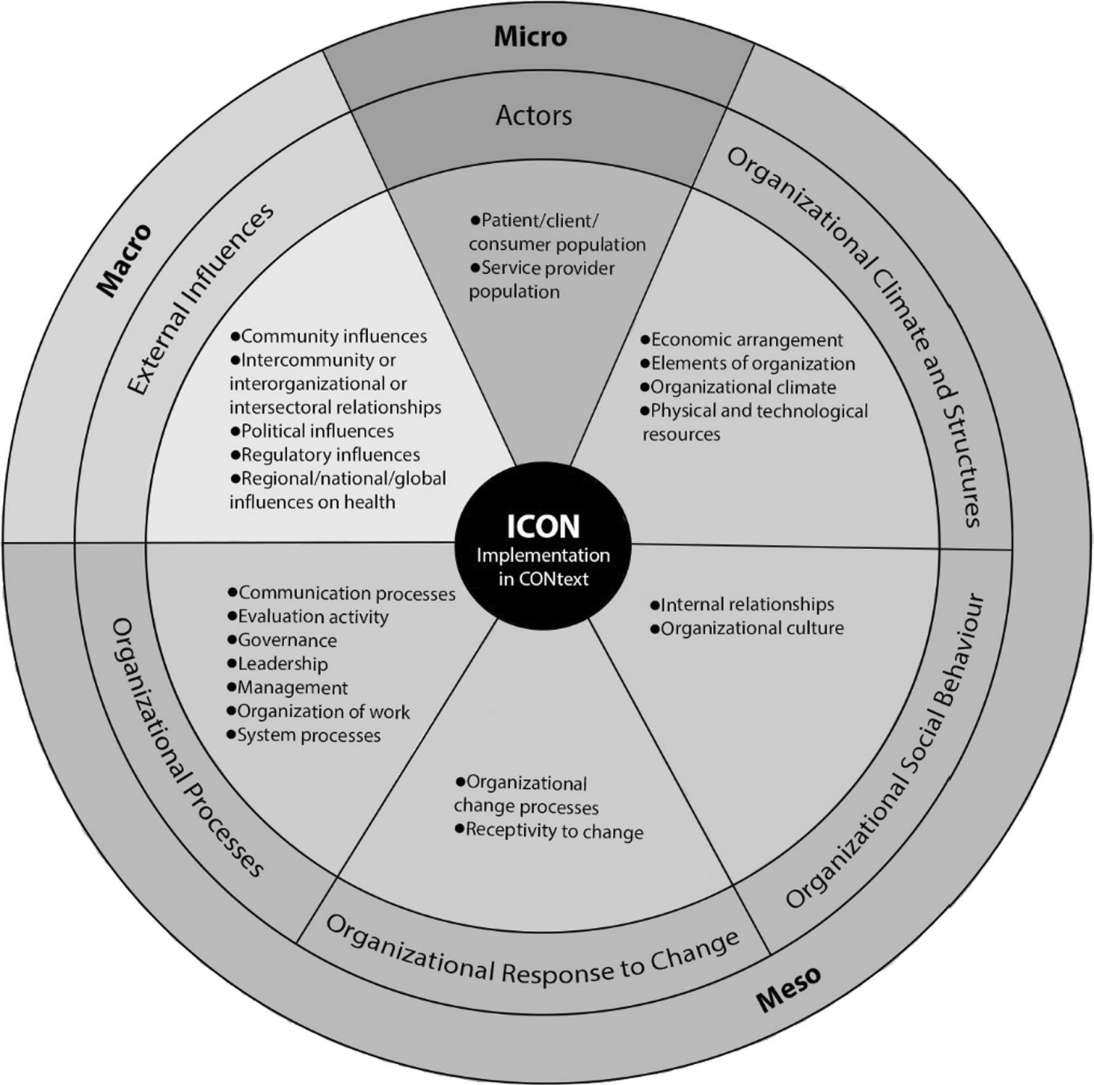
The Implementation in Context (ICON) Framework. The outer circle represents levels of context. The middle circle represents the 6 context domains within ICON. The inner circle represents the 22 core attributes of context by domain within ICON

The meta-synthesis reported in this paper resulted in the development of the ICON Framework (Fig. 2, Tables 1, 2, 3). In ICON, context is conceptualized in three levels: (1) micro (or individual), in which activities by groups of individuals in the clinical setting provide a contextual influence (for example, collective characteristics of patients or healthcare providers); (2) meso (or organizational), in which organizational characteristics are a contextual influence (for example, the culture of an organization); and (3) macro (external to the organization, including between organizations), in which market-type forces are at play (for example, political climate) [36]. These three levels contain six domains, 22 attributes and 108 features of context. The six context domains are: (1) actors (micro level), (2) organizational climate and structures (meso level), (3) organizational social behaviour (meso level), (4) organizational response to change (meso level), (5) organizational processes (meso level) and (6) external influences (macro level). The 22 defining attributes of context that align with these domains are listed and defined in Tables 1, 2, and 3.

**Table 1:** ICON micro level – attributes, attribute definitions and sample features

Domain	Attribute	Attribute definition	Example features*	Examples of considerations related to equity, diversity and inclusion
Actors	Patient/client/ consumer population	“Patient/client/consumer population” refers to the people receiving services. This attribute reflects the characteristics of patients/clients/consumers when considered as a group, rather than as individuals; thus all features considered for inclusion here have to be generalizable to a patient population (an attribute that could be potentially measured and aggregated)	• Patient/client/consumer demographics • Patient/client/consumer expectations/preferences	• Do patient/client/consumer’s equity factors (for example, place of residence, race, ethnicity, culture, language, indigenous identity, occupational status, gender, sex, gender identity, religious affiliations, educational level, socioeconomic status, social capital, age, disability, sexual preferences and relationships [61] or the intersectional combinations of these factors) affect their ability to access care, their experience with care, interactions with service providers, the quality of care they receive, their participation in care or the extent to which they are engaged in changing the type and quality of care that they/their community or population receive? • What are the health inequities in this community or population? • What are the relevant social determinants of health for this community or population?
	Service provider population	“Service provider population” refers to the characteristics, expertise and behaviour of the individuals working as providers of services. This attribute reflects individuals when considered as a group rather than as individuals; thus all features considered for inclusion here have to be generalizable to a service provider population	• Healthcare provider role • Professional development (continuing education) • Foundational healthcare provider education • Growth and career advancement • Skill set • Self-efficacy • Experience • Autonomy • Accountability • Adherence to code of ethics • Privacy and confidentiality obligation • Compulsion to act • Job satisfaction • Attitudes • Tolerance of ambiguity • Organizational commitment • Buy-in to change • Personal perception of liability • Economic incentive	• Do the service providers’ education, role, experience and skill impact their ability to consider inequities? • Do equity factors (for example, place of residence, race, ethnicity, culture, language, indigenous identity, occupational status, gender, sex, gender identity, religious affiliations, educational level, socioeconomic status, social capital, age, disability, sexual preferences and relationships [61] or the intersectional combinations of these factors) of service providers impact their growth and career advancement, attitudes, autonomy and job satisfaction? • What ethical, privacy and confidentiality considerations do service providers need to consider regarding equity-deprived populations?

*Example features – definitions of features are in Additional file 4

**Table 2:** ICON meso level** – **attributes, attribute definitions and sample features

Domain	Attribute	Attribute definition	Example features*	Examples of considerations related to equity, diversity and inclusion
Organizational climate and structures	Economic arrangements	“Economic Arrangements” refers to the income and expenditures relating to service delivery within organizations	• Funding model • Costs of healthcare delivery • Funding/ endowment	• Does the funding model/process consider equity? • Do the economic arrangements support accessible care for communities or populations who would otherwise not be able to afford it? • How can the funding organizations ensure that researchers/stakeholders meaningfully engage equity-deprived populations in studies or planning programs? • Does the organization ensure it is allocating and supporting programs that serve equity-deprived populations based on their needs? • Are there varied voices and perspectives in decisions regarding funding?
	Elements of organizations	“Elements of organizations” refers to the characteristics of units and organizations. This attribute is akin to sociodemographic characteristics for individuals	• Type of ownership • Location • Organizational size • Setting/type of environment • Physical structures • Programs (differentiation)	• Does the type of ownership affect what care is offered and accessible and the quality of care provided? • Does the location of the organization (for example, rural/urban areas, low- or high-income communities, distance of setting from the communities or populations served) affect health equity? • Are the healthcare services accessible to communities or populations with different needs (for example, are there ramps? Do they have flexible opening times? Are they accessible by public transit)?
	Organizational climate	“Organizational climate” refers to shared recurring patterns of behaviour, attitudes and feelings which characterize life in that organization [39]	• Climate (atmosphere) • Team climate • Conflict	• Is the health services team diverse and inclusive? • Is the social climate (atmosphere) welcoming to all? • Does the organization encourage self-reflection on unconscious bias toward equity-deprived populations? • Does the organization encourage tolerance and open discussion of different perspectives?
	Physical and technological resources	“Physical and technological resources” refers to physical structures and technological resources of an organization that are required to deliver services	• Space • Equipment and supplies • Technology • Online resources • Evidence-based resources • Documentation • Reminders	• Are the physical and technological resources accessible (for example, formats that are readable; are there alternative for persons with low vision)? • What accommodations might be needed to make resources accessible? • Are information materials available in different languages? • Do consumers have input or co-create resources?
Organizational social behaviour	Internal relationships	“Internal relationships” refers to the ways in which two or more people or groups within an organization regard and behave toward each other [40]	• Social networks • Social capital • Partnerships (collaborative practices)	• Does the organization encourage self-reflection of unconscious bias toward equity-deprived populations? Do they provide training for self-reflection and self-reflection of unconscious bias? • Does the organization encourage tolerance and open discussion of different perspectives? • Are patients/clients/consumers included as stakeholders in care and service decisions, and policy-making and research? • Does the organization have trusting relationships with equity-deprived populations?
	Organizational culture	“Organizational culture” refers to the normative beliefs and shared expectations that govern the work behaviour of an organization [41]	• Cultural norms • Shared expectations	• Does the organization encourage reflections on its own cultural norms/shared expectations? • Does the organization promote reflection on the influences of norms/expectations on service providers and patients’ behaviours? • Are there processes to co-create and encourage shared expectations?
Organizational response to change	Organizational change processes	“Organizational change processes” refers to the process of altering an organization’s strategies, processes, procedures, technologies and/or culture to improve service delivery [42]	• Formal change systems and processes • Quality improvement processes • Engagement • Champions/opinion leaders	• Are there processes to consider the needs of all stakeholders including equity-deprived populations? • Does the organization have processes to ensure inclusion of all stakeholders, including equity-deprived populations, in change processes? • Are the champions/opinion leaders representative of the diversity of the organization?
	Receptivity to change	“Receptivity to change” refers to an openness and responsiveness to ideas, impressions or suggestions, and a readiness or fit of critical features of the environment as they specifically relate to a targeted practice [43]. Receptivity to change may occur at multiple levels (for example, patients, healthcare professionals/providers, leaders and organization)	• Change culture • Tension for change • External pressure for change • Readiness for change • Compatibility • Change saturation	• Are assessments of readiness for change inclusive, and do they consider the diversity of the community or population? • Are the priorities of the service providers compatible with the priorities of the patients/ clients/consumers? • Does receptivity to change include equity considerations? • To what degree are equity factors integrated in changing care and services, policy and research conduct? For example, approaches related to actions on gender equity and health can be described as a continuum from gender unequal to gender blind to gender sensitive to gender specific and then gender transformative [62]
Organizational processes	Communication processes	“Communication processes” refers to the imparting or exchanging of information or news (for example, between professionals/ providers, patients, management, etc.) within an organization [44]	• Formal communication • Informal communication (social interactions) • Social influence • Advocacy	• Does the organization advocate for health equity? • Are communication strategies tailored to equity-deprived populations? • Does the organization co-create messages with clients/ consumers/patients to ensure that the terminology used, or the phrasing of the message, is respectful and inclusive? • Does the organization have processes to identify the individuals or partners who can co-create and impart messages to equity-deprived populations who might otherwise be underserved? • Does the organization ensure that there are communication channels that individuals can use without fear of discrimination or judgement after disclosure of being a member of an equity-deprived population (for example, anonymous lines)?
	Evaluation activity	“Evaluation activity” refers to the systematic collection of information about the activities, characteristics and outcomes of programs, services, policies or processes in an organization, to make judgements about the program/process, improve effectiveness and/or inform decisions about future development in that organization [45]	• Quality improvement monitoring • Performance Measurement • Performance feedback • Review of employee performance (staff/manager) • Patient/client/consumer feedback to staff	• Does the organization collect equity-related indicators/factors? • Does the organization evaluate gaps in access, quality of care and services, and health status amongst equity-deprived populations? • Does the organization evaluate the reach of interventions targeting equity-deprived populations? • What are the ethical considerations/obligations that organizations consider when collecting and presenting data related to equity and equity-deprived populations? • Do the equity-deprived populations have data sovereignty over their data? • Does the organization evaluate and meaningfully incorporate patients/clients/consumers/staffs/ managers' effort and perspectives in co-creating more equitable care and services? • Does the organization reflect on and learn from past experiences to facilitate building relationships with equity-deprived populations and prevent repeating mistakes that were detrimental to these populations?
	Governance	“Governance” refers to the rules, policies, systems, structures and processes by which an organization is controlled and directed [46]	• Organizational mission, goals & priorities • Organizational authority structure • Power • Standard of practice or care • Internal policies • Incentives and disincentives	• Do the organizational mission, goals and priorities consider equity? • How is power shared in the organization? • Do the standards of practice and care incorporate equity and the perspectives of patients/clients/consumers? • Are internal policies inclusive of individuals with varied needs? • Are there incentives and/or disincentives to promote equity in the workplace?
	Leadership	“Leadership” refers to the types and styles of leaders within an organization	• Leadership styles • Formal leaders • Senior leaders • Role models • Mentors	• How diverse is the leadership team? • Are there role models and mentors within the organization from diverse backgrounds?
	Management	“Management” refers to the process of dealing with or organizing things or people in an organization [47]	• Formal planning • Management support • Use of resources	• Does the organization consider equity during policy planning, work planning, strategic planning and setting annual goals? • Does the organization meaningfully engage diverse groups of individuals in formal planning? • Do the processes related to resource allocation consider equity?
	Organization of work	“Organization of work” refers to arrangement of tasks, responsibilities and resources within and between service providers working in the organization [48]. This attribute reflects individuals when considered as a group rather than as individuals, thus all features considered for inclusion here had to be generalizable to an organization	• Workload • Adequacy of staff composition • Support personnel • Teamwork • Scheduling • Workflow • Work tempo • Time	• Does the organization have individuals who provide expertise regarding equity and its integration in care and services, policy and research? • Does the organization have a diverse workforce? • Does the organization celebrate the diversity of its staff and encourage individuals to express themselves freely? • Does the organization support its staff to consider equity in their work?
	System processes	“System processes” refers to the processes required to deliver services within an organization	• Quality assurance process • Project Management • Optimizing standardization of care • Continuity of Care • Organizational training and education • Process complexity • System complexity	• Do current system processes incorporate equity? • Does the organization provide training for self-reflection and awareness of unconscious bias? • Do standards of care consider the diversity of care needs unique to each community or population? • Are there system processes in place to allow the organization to respond to circumstances that are incompatible or harmful to equity-deprived populations?

*Example features – definitions of features are in Additional file 4

**Table 3:** ICON macro level – attributes, attribute definitions and sample features

Domain	Attribute	Attribute definition	Example features*	Examples of considerations related to equity, diversity and inclusion
	Community influences	“Community influences” refers to influences from the general public or other organizations on the decision-making and behaviour of organizations and the individuals (as an aggregated group) within these organizations	• Public influences • Peer organizational pressure	• Does the organization promote open dialogue with the communities or populations it serves? • Does the organization build and maintain trusting relationships with the communities or populations it serves? • Are the community influences representative of diverse populations?
	Intercommunity/ interorganizational/ intersectoral relationships	“Intercommunity/interorganizational/intersectoral relationships” refers to the interactions or partnerships between different communities, organizations or sectors	• Intersectoral collaboration • Community health outreach • Coordinated action	• Do stakeholders from other sectors (for example, city planners) partner with the organization to impact the health of individuals, especially equity-deprived populations? • Do community organizations or communities collaborate with the organization to improve health equity and well-being? • Do the actions and goals of the organization align with their community partners?
	Political influences	“Political Influences” refers to influences relating to government or public affairs and their effect on organizations and individuals (as an aggregated group) within these organizations	• Politics • Political climate • Complexity of the broader sociopolitical environment	• Are there laws and policies requiring consideration of equity? • Does the organization lobby for reform of laws and policies that impact health equity?
	Regulatory influences	“Regulatory influences” refers to influences relating to regulatory bodies, created on the basis of a legal mandate or legislation (for example, providing and enforcing on adequate standards for health and safety in an organization) [49]	• Laws and legislation • External policies, directives, mandates and regulations • Industrial influences • Accreditation standards	• Are there laws and policies requiring consideration of equity? • Does the organization lobby for reform of laws and policies that impact health equity? • Do accreditation standards include criteria relevant to equity?
	Regional/national/global influences on health	“Regional/national/global influences on health” refers to regional, national and worldwide influences on organizations and individuals (as an aggregated group) within these organizations. For example, epidemics, pandemics, endemics, outbreaks, natural disasters, climate change and war	• Epidemics/pandemics/endemics/outbreaks • Natural disasters • Climate change	• Is there a process for determining which groups are more vulnerable to health inequities caused by epidemics, pandemics, endemics, outbreaks, natural disasters and/or climate change? • Do organizational processes for distributing relief or support in response to regional/national/global health influences consider equity? • Does the organization fulfil its role and responsibilities in decreasing its impact on climate change?

*Example features – definitions of features are in Additional file 4

These contextual attributes are interconnected and have potential to directly or indirectly influence or modify the use of evidence by healthcare providers, policy-makers and recipients of care. For each of these context attributes, we identified several features (examples) that further elaborate on the meaning of the attributes. ICON is illustrated in Fig. 2. ICON attributes and their definitions, as well as sample features and equity, diversity and inclusion (EDI) considerations of each attribute, for the micro, meso and macro levels are presented in Tables 1, 2, and 3, respectively. Identification and definitions of all 108 features contained in ICON are presented in alphabetical order in Additional file 4.

### Description of ICON by level and domain of context

The broad structure of ICON is reflective of the organizational literature [37, 38], where context is examined from the perspective of three levels: (1) micro (or individual), (2) meso (or organizational) and (3) macro (external to the organization, including between organizations).

#### Micro (individual) level of context

The micro level of ICON is summarized below; see Table 1 and Additional file 4 for additional details on the attributes and features contained within the micro level of ICON. This level of ICON contains a single context domain: actors.

##### Domain 1: Actors

The actors domain comprises two attributes: (1) patient/client/consumer population and (2) service provider (healthcare professional) population. Both attributes reflect individuals when considered as a group rather than as individuals. The patient/client/consumer population attribute refers to the characteristics of a group of individuals receiving care/services. We identified two unique features that illustrate this attribute: patient/client/consumer demographics (who they are) and patient/client/consumer expectations/preferences (what they want). The second attribute that comprises the actor domain is the service provider population. This attribute refers to the collective characteristics, expertise and behaviour of the individuals working as providers of services. We identified 19 unique features within this attribute; example features include healthcare provider role, experience and attitudes.

#### Meso (organizational) level of context

The meso level of ICON is summarized next; see Table 2 and Additional file 4 for additional details on the attributes and features contained within the meso level of ICON. This level of ICON contains four of the six context domains: (1) organizational climate and structures, (2) organizational social behaviour, (3) organizational response to change and (4) organizational processes.

##### Domain 2: Organizational climate and structures

The organizational climate and structures domain comprises four attributes: (1) economic arrangements (*n* = 3 features), (2) elements of organizations (*n* = 6 features), (3) organizational climate (*n* = 3 features) and (4) physical and technological resources (*n* = 7 features). The attribute economic arrangements refers to the income and expenditures relating to service delivery within organizations; example features include funding models and healthcare delivery costs. The attribute elements of organizations refers to the characteristics of units and organizations. This attribute is akin to sociodemographic characteristics for individuals. Example features within this attribute include type of ownership (for example, public, private or faith-based) and organizational size.

The attribute organizational climate refers to shared recurring patterns of behaviour, attitudes and feelings that characterize life in that organization [39]; example features include team climate and conflict. Lastly, the attribute physical and technological resources refers to an organization’s physical structures and technological resources that are required to deliver services; example features include space and online resources.

##### Domain 3: Organizational social behaviour

The organizational social behaviour domain comprises two attributes: (1) internal relationships (*n* = 3 features) and (2) organizational culture (*n* = 2 features). The attribute internal relationships refers to the ways in which two or more people or groups within an organization regard and behave toward each other [40]; example features include social networks and social capital. The attribute organizational culture refers to the normative beliefs and shared expectations that govern the work behaviour in an organization [41]; example features include cultural norms and shared expectations.

##### Domain 4: Organizational response to change

The organizational response to change domain comprises two attributes: (1) organizational change processes (*n* = 4 features) and (2) receptivity to change (*n* = 6 features). The attribute organizational change processes refers to the process of altering an organization’s strategies, processes, procedures, technologies and/or culture to improve service delivery [42]; example features include formal change systems and processes and engagement. The attribute receptivity to change refers to openness and responsiveness to ideas, impressions or suggestions, and readiness or fit of critical features of the environment as they specifically relate to a targeted practice [43]. Receptivity to change may occur at multiple levels (for example, patients, healthcare professionals/providers, leaders and organization). Example features of receptivity to change included in ICON are change culture and readiness for change.

##### Domain 5: Organizational processes

The organizational processes domain comprises seven attributes: (1) communication processes (*n* = 4 features), (2) evaluation activity (*n* = 5 features), (3) governance (*n* = 6 features), (4) leadership (*n* = 5 features), (5) management (*n* = 3 features), (6) organization of work (*n* = 8 features) and (7) system processes (*n* = 7 features). The attribute communication processes refers to imparting of, or exchanging, information or news (for example, between healthcare professionals/providers, patients and management) within an organization [44]; example features include formal communication and social influence. The attribute evaluation activity refers to the systematic collection of information about the activities, characteristics and outcomes of programs, services, policies or processes in an organization to make judgements about the program/process, improve effectiveness and/or inform decisions about future development in that organization [45]. Example features of evaluation activity include quality improvement monitoring and performance measurement. The attribute governance refers to the rules, policies, systems, structures and processes by which an organization is controlled and directed; example features include organizational mission, goals and priorities, and incentives and disincentives [46]. The attribute leadership refers to the types and styles of leaders within an organization; example features include formal leaders as well as role models and mentors. The attribute management refers to the process of dealing with or organizing things or people in an organization [47]; example features include formal planning and the use of resources. The attribute organization of work refers to the arrangement of tasks, responsibilities and resources within and between service providers working in the setting [48]. Example features within this attribute include workload and teamwork. Lastly, the attribute system processes refers to the processes required to deliver services within an organization; example features include quality assurance and continuity of care.

#### Macro (external) level of context

The macro level of ICON is summarized next; see Table 3 and Additional file 4 for additional details on the attributes and features contained within the macro level of ICON. This level of ICON contains a single domain: external influences.

##### Domain 6: External influences

The external influences domain comprises five attributes: (1) community influences (*n* = 2 features), (2) intercommunity/interorganizational/intersectoral relationships (*n* = 3 features), (3) political influences (*n* = 3 features), (4) regulatory influences (*n* = 4 features) and (5) regional/national/global health influences (*n* = 3 features). External influences can affect organizations as a complete entity, or the activity and functions of individuals (as an aggregated group, for example, teams or professional designations) present within an organization. The attribute community influences refers to influences from society at large; example features include public influences and peer organizational pressure. The attribute intercommunity/interorganizational/intersectoral relationships refers to the interactions or partnerships between different communities, organizations or sectors; example features include intersectoral collaboration and community health outreach. The attribute political influences refers to influences from government or public affairs; example features include politics and the political climate. The attribute regulatory influences refers to influences relating to regulatory bodies, created based on a legal mandate or legislation (for example, providing and enforcing standards for health and safety in an organization) [49]; example features include laws and legislation and the external policies, directives, mandates and regulations. Lastly, the attribute regional/national/global health influences refers to regional, national and worldwide influences. Example features within this attribute include epidemics/pandemics/endemics/outbreaks and climate change.

## Discussion

The importance of context in influencing implementation success is well established [1–4, 13, 50–53]. Comprehensive guidance for implementers and researchers on what comprises context for implementation purposes is necessary but has been lacking. Such guidance is key to informing implementation intervention design and testing, context measurement and evaluation. The ICON Framework addresses this need, having been founded on a rigorous and systematic approach, involving an incremental series of complementary studies [27–31], to develop a comprehensive understanding of context for implementation purposes.

### How ICON advances implementation science

ICON is the only implementation meta-framework solely dedicated to the role of context in implementation that is available to guide implementation efforts. Context is included in other implementation frameworks, for example: Diffusion of Innovations Theory [19]; Promoting Action on Research Implementation in Health Services Framework [20]; Integrated-Promoting Action on Research Implementation in Health Services [21]; Ottawa Model of Research Use [22]; Knowledge-to-Action Framework (KTA) [23]; Consolidated Framework for Implementation Research [24, 54]; Exploration, Preparation, Implementation, Sustainment Framework [25]; and Tailored Implementation in Chronic Diseases Checklist [26]. There are, however, important inconsistencies between these frameworks in how context is defined conceptually. For instance, while later frameworks such as the Integrated-Promoting Action on Research Implementation in Health Services [21], Consolidated Framework for Implementation Research [24, 54]; and the Exploration, Preparation, Implementation, Sustainment Framework [25] make explicit distinctions between inner and outer contexts, earlier conceptualizations such as Diffusion of Innovations Theory [19], Promoting Action on Research Implementation in Health Services [20] and Knowledge to Action Framework [23] do not. These frameworks are also inconsistent and incomplete with respect to the attributes of context they include. In contrast, some attributes are represented in more than one framework (for example, leadership and culture), and many attributes are unique to a single framework (for example, continuing educational system is only specified in the Tailored Implementation in Chronic Diseases Checklist [26]). Other attributes, based on ICON, are missing entirely from these frameworks (for example, attribute – elements of organizations; example features – facility size, location and type of ownership). ICON is the only synthesis and framework to date to comprehensively capture in a single place all core attributes of context that are relevant to implementation by healthcare professionals in clinical practice.

The ICON Framework differs from other implementation frameworks in several ways. First, the ICON Framework includes a main list of domains, attributes and features by level of context, providing much-needed conceptual clarity about context. Second, the ICON Framework provides a comprehensive representation of the core attributes of context relevant to implementation in healthcare. Finally, the ICON Framework includes attributes and features not previously identified; approximately half the features are not included in current implementation frameworks [28, 29]. Such a meta-framework of context with a high level of detail about the domains, attributes and features of context for implementation has not been developed previously. Thus, ICON substantially expands on previous understanding of context for implementation in healthcare.

### How to use ICON

The ICON Framework guides knowledge users (for example, implementers) and implementation researchers seeking to design, deliver and evaluate implementation interventions. The ICON Qualitative Screening Tool (Additional file 5) was developed to complement ICON and enable prioritization of which core context attributes to measure, dependent on the evidence being implemented and the implementation circumstances. The screening tool contains seven questions addressing the six ICON domains and any other contextual factors that may influence implementation efforts. For each domain-related question, prompts concerning attributes within the respective domain are provided for use, as appropriate. The questions should be framed according to the implementation intervention being considered. Pre-tested by 10 knowledge users, when administered in interview format the tool took, on average, 20 min to complete. Feedback about the tool indicated it raised awareness about aspects of context that had not previously been considered to influence implementation efforts, highlighting areas that needed closer attention to improve the likelihood of implementation success. This screening tool was designed as a practical means for implementation teams to develop a broad understanding of the specific context in which they are working so they can identify areas in which more detailed exploration and measurement would be useful. The brief tool can readily be self-administered or administered through individual or focus group interviews of key informants. The use of the tool then enables implementation teams to invest resources in the measurement of aspects of context important to their specific implementation effort. It is important to acknowledge that context is dynamic. Therefore, while assessing each context attribute individually at a single point in time with the ICON screening tool will provide valuable information to help support implementation, it is important to concurrently consider the dynamic interactions between these context attributes that will not be captured when components are assessed independently of one another. It is also important to re-assess context periodically with the screening tool due to its temporal nature.

Implementation teams composed of knowledge users, implementers and/or patients/clients/consumers can use both the qualitative screening tool and the EDI considerations outlined in Table 1 to capture diverse perspectives pertaining to the implementation of care or services (for example, facilitators and barriers) and the priorities and outcomes that members of implementation teams seek to achieve. The EDI considerations can help teams to recognize the communities and populations whose perspectives are being excluded, whose voices are being amplified and the contextual factors that can be modified to ensure meaningful inclusion of equity-deprived communities and populations during implementation. Some of the EDI considerations are intended to facilitate reflexivity amongst teams regarding intersectionality pertaining to patients/clients/consumers, service providers and the diversity of their team composition. Furthermore, the EDI considerations for the ICON attributes and factors can be a way for organizations to evaluate their progress and stay accountable, which could prevent the criticism of performative EDI described by other scholars [55].

The Alliance for Health Policy and Systems Research reported four elements that should receive greater attention: trust and power, engaging communities and focusing on rights, looking beyond the health sector and considering factors beyond national boundaries [56]. These elements are present in ICON. Trust and power are represented in the internal relationship attribute (one example feature being partnership) and in the governance attribute (one example feature being power). Engaging communities and focusing on rights is represented by the intercommunity/interorganizational/intersectoral relationships attribute (two of the example features being community health outreach and coordinated action). Looking beyond the health sector is represented in the intercommunity/interorganizational/intersectoral relationships attribute (one example feature being intersectoral collaboration) and the political influences attribute (one example feature being the complexity of the broader sociopolitical environment). Considering factors beyond national boundaries is represented in the regional/national/global influences on health attribute (one example feature being climate change). In keeping with the Alliance for Health Policy and Systems Research stance [56], we emphasized in a response to commentaries [57] written on one of the studies [29] that informed the ICON Framework, that the ICON Framework has detailed descriptions of the context attributes at the macro level (for example, regulatory, and legislative standards). One of the commentators [58] reported that this level of detail is beneficial, as these context attributes and features are often under-assessed and under-developed aspects of context.

### Next steps

Having advanced conceptual clarity and detail on context by defining it and by identifying its core domains, attributes and features – as depicted in the ICON Framework – the next logical steps to advance this work are currently underway to: (1) identify and evaluate measures that currently exist for each of the ICON Framework core 22 defining attributes, (2) evaluate the psychometric and pragmatic properties of the identified measures and (3) create a publicly accessible registry of these measurement tools, and assessments. The registry will contain a built-in decision-making algorithm to assist implementation teams in selecting the most appropriate measure(s) for their specific implementation effort. Future work should also investigate the transferability of ICON to regions and groups not reflected in the current data used to develop ICON, for example, the Global South, Indigenous communities, and non-English-speaking communities.

### Limitations

Limitations of this work need to be acknowledged. While our approach involved a series of complementary studies to examine context rigorously and comprehensively from different perspectives and using different methodologies, followed by a rigorous meta-synthesis to produce the ICON Framework, it is possible that some attributes and/or features of context were not identified. A design element that likely constrained the attributes and features represented in ICON is that the published research we drew on, and the participants included in our research, largely reflected the Global North (for example, high-resourced settings such as North America, Europe and Australia). As such, context attributes and features that might be unique to the Global South (for example, low- and middle-resources settings in Asia, Africa, Oceania and Latin America) may have been omitted from ICON. Finally, given the more recent focus on equity and intersectionality in implementation science [59, 60], intersectional attributes related to context are likely not adequately reflected in the current version of ICON. The concept of intersectionality is not well represented in the literature we analysed in our concept analysis [27], and it was not specifically explored, nor was it identified by participants in the two interview studies [28, 29]. However, an intersectional lens can be applied to all aspects of the ICON Framework. Specifically, implementers could actively explore intersectional considerations for each attribute. Finally, ICON is a framework, not a theory; therefore, no associations between its domains/attributes/features are inferred or can be made.

## Conclusion

ICON provides comprehensive and critically needed clarity of context as a concept and its domains, attributes and features. As such, it advances implementation science and the potential to influence healthcare professionals’ use of research in clinical practice. The ICON Framework consolidates and unifies the core attributes of context relevant to implementation derived from the published literature, and it embraces perspectives from a wide range of healthcare professionals, as well as tacit knowledge from health system stakeholders internationally. It is now time to identify, and develop where needed, assessment tools to measure the ICON defining attributes that will enable optimal tailoring of implementation intervention design and delivery, better interpretation of the effects of implementation interventions, and pragmatically guide knowledge users in their implementation efforts. ICON is the first framework of its kind, and it is poised to advance the practice and science of implementation substantially. We invite readers of this manuscript to use ICON in their implementation efforts and report on their experiences with it.

### Supplementary Information

**Additional file 1.** Enhancing transparency in reporting the synthesis of qualitative research: the ENTREQ statement.

**Additional file 2.** Databases searched for concept analysis on context and the disciplines they cover.

**Additional file 3.** Rankings from Knowledge Utilization Colloquium 2019 on ICON Framework.

**Additional file 4.** Features of context in ICON.

**Additional file 5.** ICON Qualitative Screening Tool.

## Data Availability

The ICON features, the ranking of ICON attributes from the *2019 Knowledge Utilization Colloquium* and the ICON screening tool are provided as additional files. The datasets used and/or analysed during the current study are available from the corresponding author on reasonable request.

## Abbreviations

ENTREQ: Enhancing Transparency in Reporting the Synthesis of Qualitative Research Checklist
ICON framework: Implementation in Context Framework
EDI: Equity, diversity and inclusion
