# Supplementary material for: The Implementation in Context (ICON) Framework: A meta-framework of context domains, attributes and features in healthcare
Source: Health Res Policy Syst. 2023 Aug 7;21:81. doi: 10.1186/s12961-023-01028-z (PMC10408185; doi:10.1186/s12961-023-01028-z)
Supplement: Supplementary file 1 — Additional file 1. Enhancing transparency in reporting the synthesis of qualitative research: the ENTREQ statement. [file 12961_2023_1028_MOESM1_ESM.docx]

**Additional File 1**

**Enhancing transparency in reporting the synthesis of qualitative research: the ENTREQ statement**

| **No** | **Item** | **Guide and description** | **Page Number/Section** |
| --- | --- | --- | --- |
| 1 | Aim | State the research question the synthesis addresses. | Page 5 (Background) |
| 2 | Synthesis methodology | Identify the synthesis methodology or theoretical framework which underpins the synthesis and describe the rationale for choice of methodology (e.g., meta-ethnography, thematic synthesis, critical interpretive synthesis, grounded theory synthesis, realist synthesis, meta-aggregation, meta-study, framework synthesis). | Page 8-10 (Methods) |
| 3 | Approach to searching | Indicate whether the search was pre-planned (comprehensive search strategies to seek all available studies) or iterative (to seek all available concepts until they theoretical saturation is achieved). | Page 6-8 (Methods) |
| 4 | Inclusion criteria | Specify the inclusion/exclusion criteria (e.g., in terms of population, language, year limits, type of publication, study type). | Page 6-8 (Methods) and more details are present in the studies we cited. |
| 5 | Data sources | Describe the information sources used (e.g., electronic databases (MEDLINE, EMBASE, CINAHL, psycINFO, Econlit), grey literature databases (digital thesis, policy reports), relevant organisational websites, experts, information specialists, generic web searches (Google Scholar) hand searching, reference lists) and when the searches conducted; provide the rationale for using the data sources. | Page 6-8 (Methods) and more details are present in the studies we cited. |
| 6 | Electronic Search strategy | Describe the literature search (e.g., provide electronic search strategies with population terms, clinical or health topic terms, experiential or social phenomena related terms, filters for qualitative research, and search limits). | Page 6-8 (Methods) and more details are present in the studies we cited. |
| 7 | Study screening methods | Describe the process of study screening and sifting (e.g., title, abstract and full text review, number of independent reviewers who screened studies). | Page 6-8 (Methods) and more details are present in the studies we cited. |
| 8 | Study characteristics | Present the characteristics of the included studies (e.g., year of publication, country, population, number of participants, data collection, methodology, analysis, research questions) | Page 6-8 (Methods) and more details are present in the studies we cited. |
| 9 | Study selection results | Identify the number of studies screened and provide reasons for study exclusion (e.g., for comprehensive searching, provide numbers of studies screened and reasons for exclusion indicated in a figure/flowchart; for iterative searching describe reasons for study exclusion and inclusion based on modifications to the research question and/or contribution to theory development) | Page 6-8 (Methods) and more details are present in the studies we cited. |
| 10 | Rationale for appraisal | Describe the rationale and approach used to appraise the included studies or selected findings (e.g., assessment of conduct (validity and robustness), assessment of reporting (transparency), assessment of content and utility of the findings). | Not applicable |
| 11 | Appraisal items | State the tools, frameworks and criteria used to appraise the studies or selected findings; reviewer developed tools; describe the domains assessed: research team, study design, data analysis and interpretations, reporting). | Not applicable |
| 12 | Appraisal process | Indicate whether the appraisal was conducted independently by more than one reviewer and if consensus was required. | Not applicable |
| 13 | Appraisal results | Present results of the quality assessment and indicate which articles, if any, were weighted/excluded based on the assessment and give the rationale. | Not applicable |
| 14 | Data extraction | Indicate which sections of the primary studies were analysed and how were the data extracted from the primary studies? (e.g., all text under the headings “results /conclusions” were extracted electronically and entered into a computer software). | Page 8-10 (Meta-synthesis Protocol - The Current Study) |
| 15 | Software | State the computer software used, if any. | Page 9 (Meta-synthesis Protocol - The Current Study) |
| 16 | Number of reviewers | Identify who was involved in coding and analysis. | Page 8-10 (Meta-synthesis Protocol - The Current Study) |
| 17 | Coding | Describe the process for coding of data (e.g., line by line coding to search for concepts). | Page 8-10 (Meta-synthesis Protocol - The Current Study) |
| 18 | Study comparison | Describe how were comparisons made within and across studies (e.g., subsequent studies were coded into pre-existing concepts, and new concepts were created when deemed necessary). | Page 8-10 (Meta-synthesis Protocol - The Current Study) |
| 19 | Derivation of themes | Explain whether the process of deriving the themes or constructs was inductive or deductive | Page 8-10 (Meta-synthesis Protocol - The Current Study) |
| 20 | Quotations | Provide quotations from the primary studies to illustrate themes/constructs and identify whether the quotations were participant quotations of the author’s interpretation. | Not applicable |
| 21 | Synthesis output | Present rich, compelling and useful results that go beyond a summary of the primary studies (e.g., new interpretation, models of evidence, conceptual models, analytical framework, development of a new theory or construct). | Page 11-17 (Results, Figure 2 and Tables1-3) |

Adapted from: Tong A, Flemming K, McInnes E, Oliver S, Craig J. Enhancing transparency in reporting the synthesis of qualitative research: ENTREQ. BMC Med Res Methodol. 2012 Dec;12(1):1-8.
