# Supplementary material for: The Implementation in Context (ICON) Framework: A meta-framework of context domains, attributes and features in healthcare
Source: Health Res Policy Syst. 2023 Aug 7;21:81. doi: 10.1186/s12961-023-01028-z (PMC10408185; doi:10.1186/s12961-023-01028-z)
Supplement: Supplementary file 2 — Additional file 2. Databases searched for concept analysis on context and the disciplines they cover. [file 12961_2023_1028_MOESM2_ESM.doc]

**Additional File 2**

**Databases searched for concept analysis on context and the disciplines they cover**

| Database | Disciplines Covered |
| --- | --- |
| Business Source Complete, Ebsco | Business, Civil Engineering, Computer Engineering, Computer Science, Economics, Electrical Engineering, Engineering, Health Information, Law, Mechanical Engineering, Public Administration, Software Engineering^1^ |
| CINAHL (Cumulative Index to Nursing and Allied Health Literature),  EbscoHost | Nursing and Allied Health^2^ |
| Cochrane Central Register of Controlled Trials (CENTRAL) | Healthcare related clinical trials^3^ |
| Cochrane Database of Systematic Reviews | Systematic review of research in healthcare and health policy^4^ |
| Dissertations and Theses Database, ProQuest | Multidisciplinary and international database for theses and dissertations^5^ |
| EMBASE, OvidSP | Medicine, Nursing & Health Professions, Behavioral Sciences, Basic Sciences  Humanities & Technologies  Agriculture and Food Sciences  Engineering^6^ |
| MEDLINE(R) and In‐Process and other non‐indexed citations, OvidSP | Biomedicine, allied health fields, biological and physical sciences, humanities, and information science as they relate to medicine and healthcare^7^ |
| NHS Economic Evaluation Database (NHS EED) | Economic evaluations in healthcare, cost effectiveness of health technologies^8,9^ |
| PAIS (Public Affairs International), Proquest | Issues in the public debate from various international sources such as journal articles, books, government documents, grey literature etc. These subjects include the following: Atomic Age; China's cultural revolution; Civil rights movement; Cold War; Communism, Socialism, Nationalism; Great Depression; Israeli-Palestinian conflict; Korean War  McCarthyism; Prohibition; Religion and public schools; Space exploration; United Nations and League of Nations; Vietnam conflict; Watergate; Women's movement; World Wars I and II^10^ |
| PsycINFO, OvidSP | Abnormal Psychology; Applied Psychology  Behavioral Psychology; Business & Economics; Child Development Psychology  Clinical Psychology; Cognitive Psychology  Community Psychology; Education Psychology; Experimental Psychology  Medical Law; Ethics & Other Humanities  Neuropsychology; Neuroscience  Perception; Psychiatry; Psycholinguistics  Psychology; Psychotherapy; Rehabilitation  Social Psychology; Social Work; Sociology  Speech; Language & Hearing^11^ |
| PubMed | Biomedicine and health fields, and related disciplines (e.g., life sciences, behavioral sciences, chemical sciences, and bioengineering)^12^ |
| Science Citation Index and Social Sciences Citation Index (ISI Web of Knowledge) | a) Science Citation Index: Multidisciplinary and broad database that indexes disciples from the sciences, social sciences, and humanities^13^  b) Social Sciences Citation Index: social science journals across 55 disciplines such as communication, education, geography, history & philosophy of the social sciences, law, political science, public health, social issues & sociology^14^ |
| Web of Science, Conference Proceedings Citation Index‐ Science (ISI Web of Knowledge) | Multidisciplinary and broad database for conference proceedings in the science, social science, and humanities disciplines^15^ |
| WorldCat (an international catalogues of books) | Multidisciplinary and international catalogues of books and other library resources^16^ |

**References**

1. University of Victoria. Business Source Complete (EBSCO). 2023; <https://webapp.library.uvic.ca/databases/details.php?id=144>. Accessed May 23, 2023.

2. EBSCO Information Services. CINAHL Database. 2023; <https://www.ebsco.com/products/research-databases/cinahl-database>. Accessed May 23, 2023.

3. Cochrane. Cochrane Central Register of Controlled Trials (CENTRAL). 2023; <https://www.cochranelibrary.com/central/about-central>. Accessed May 23, 2023.

4. Cochrane. About Cochrane Reviews. 2023; <https://www.cochranelibrary.com/about/about-cochrane-reviews>. Accessed May 23,, 2023.

5. ProQuest. ProQuest Dissertations & Theses Global. 2023; <https://about.proquest.com/en/products-services/pqdtglobal/>. Accessed May 23, 2023.

6. Wolters Kluwer. About Ovid. 2023; <https://www.wolterskluwer.com/en/solutions/ovid/about>. Accessed May 23, 2023.

7. National Library of Medicine. MEDLINE® 2023 Database Guide. 2023; <https://ospguides.ovid.com/OSPguides/medline.htm>. Accessed May 23, 2023.

8. Nixon J, Stoykova B, Christie J, Glanville J, Kleijnen J, Drummond M. NHS Economic Evaluation Database for healthcare decision makers. *BMJ.* 2000;321(7252):32.

9. Centre for Reviews & Dissemination. NHS Economic Evaluation Database Guide. 2023; <https://ospguides.ovid.com/cleeddb.htm>. Accessed May 23, 2023.

10. ProQuest. PAIS® Index. 2023; <https://about.proquest.com/en/products-services/paisarc-set-c/>, 2023.

11. Wolters Kluwer. APA PsycInfo®. 2023; <https://www.wolterskluwer.com/en/solutions/ovid/apa-psycinfo-139>. Accessed May 24, 2023.

12. National Library of Medicine. PubMed Overview. 2022; <https://pubmed.ncbi.nlm.nih.gov/about/>. Accessed May 24, 2023.

13. The Trustees of Princeton University. Science Citation Index. 2023; <https://library.princeton.edu/resource/title/science-citation-index>. Accessed May 24, 2023.

14. Clarivate. Social Sciences Citation Index. 2020; <http://wokinfo.com/products_tools/multidisciplinary/webofscience/ssci/>. Accessed May 24, 2023.

15. Clarivate. Web of Science Conference Proceedings Citation Index Evaluation Process and Selection Criteria. 2023; <https://clarivate.com/products/scientific-and-academic-research/research-discovery-and-workflow-solutions/web-of-science/core-collection/editorial-selection-process/web-of-science-conference-proceedings-citation-index-evaluation-process-and-selection-criteria/>. Accessed May 24, 2023.

16. Worldcat. About WorldCat.org. 2023; <https://www.worldcat.org/about>. Accessed May 24, 2023.
