# Supplementary material for: The Implementation in Context (ICON) Framework: A meta-framework of context domains, attributes and features in healthcare
Source: Health Res Policy Syst. 2023 Aug 7;21:81. doi: 10.1186/s12961-023-01028-z (PMC10408185; doi:10.1186/s12961-023-01028-z)
Supplement: Supplementary file 3 — Additional file 3. Rankings from Knowledge Utilization Colloquium 2019 on ICON Framework. [file 12961_2023_1028_MOESM3_ESM.docx]

**Additional File 3**

**Rankings from Knowledge Utilization Colloquium 2019 on ICON Framework**

| **ICON Level** | **ICON Domain** | **ICON Attribute** | **Median Ranking** | |
| --- | --- | --- | --- | --- |
|  |  |  | Researchers | Knowledge Users |
| Micro | Actors | Patient/Client/Consumer Population | 1 | 1 |
|  |  | Service Provider Population | 1 | 1 |
| Meso | Organizational Climate and Structures | Organizational Climate | 1 | 1 |
|  |  | Elements of Organizations | 1 | 2 |
|  |  | Economic Arrangements | 3 | 1 |
|  |  | Physical and Technological Resources | 2 | 2 |
|  | Organizational Social Behaviour | Internal Relationships | 1 | 1 |
|  |  | Organizational Culture | 1 | 1 |
|  | Organizational Response to Change | Receptivity to Change | 1 | 1 |
|  |  | Organizational Change Processes | 1 | 1 |
|  | Organizational Processes | Governance | 2 | 2 |
|  |  | Management | 3 | 1 |
|  |  | Organization of Work | 1 | 1 |
|  |  | System Processes | 1 | 1 |
|  |  | Evaluation Activity | 1 | 1 |
|  |  | Leadership | 1 | 1 |
|  |  | Communication Processes | 1 | 1 |
| Macro | External Influences | Intercommunity/Interorganizational/Intersectoral Relationships | NA | NA |
|  |  | Community Influences | 3 | 3 |
|  |  | Political Influences | 1 | 1 |
|  |  | Regulatory Influences | NA | NA |
|  |  | Regional/National/Global Health Influences | NA | NA |

NA – added post this assessment

**Question Asked:**

When designing an implementation intervention, how important is it to assess each of the following ICON attributes of context?

1-Very important 2-Important 3-Moderately important 4-Slightly important 5-Not important

**Note:**

Some features were reorganized and labels (names) of attributes were adjusted following this assessment. This table above uses the final labels as presented in the current version of ICON.
