# Supplementary material for: The Implementation in Context (ICON) Framework: A meta-framework of context domains, attributes and features in healthcare
Source: Health Res Policy Syst. 2023 Aug 7;21:81. doi: 10.1186/s12961-023-01028-z (PMC10408185; doi:10.1186/s12961-023-01028-z)
Supplement: Supplementary file 4 — Additional file 4. Features of context in ICON. [file 12961_2023_1028_MOESM4_ESM.docx]

**Additional File 4**

**Features of context in ICON**

**Table 1: Features of Context in ICON (Micro Level)**

| **Attribute** | **Example Features** | **Feature Description** |
| --- | --- | --- |
| **Domain: Actors** | | |
| Patient/Client/Consumer Population | Patient/Client/Consumer Demographics | Characteristics of the population receiving care or service including (but not limited to) their collective age, sex, gender, socio-economic status, education, marital status, cultural background, and other intersectionality categories, (e.g., race, class, sexual orientation, religion), health status (number of illnesses or comorbidities, patient acuity, illness severity, etc.), common illness/health trajectories, health inequities and vulnerable groups. |
|  | Patient/Client/Consumer Expectations/  Preferences | This includes the range of expressed or implicit requests, desires, and demands of patients involved in an encounter. This also includes a range of desires and demands of patients' families when acting in the role of decision-maker/surrogate. |
| Service Provider Population | Healthcare Provider Role | Expectations, both formal and informal, associated with a given healthcare occupation |
|  | Professional Development (Continuing Education) | Education and training undertaken as part of the normal course of a service provider’s professional activities. |
|  | Foundational Healthcare Provider Education | The core training required for registration/certification, often occurring in college or university (e.g., medical, nursing, physiotherapy, occupational therapy, social work programs, personal support worker), which defines a particular healthcare provider role. |
|  | Growth and Career Advancement | The extent to which service providers’ value and use opportunities for their own professional growth^1^. |
|  | Skill Set | Particular technical competencies and abilities that typify a specific service provider role. For example, taking a blood pressure, suturing, critical appraisal skills. |
|  | Self-Efficacy | Confidence in one’s own skills and performance^1^. |
|  | Experience | Having knowledge or skills in a particular field gained over a period of time. |
|  | Autonomy | The amount of freedom a service provider group has to make decisions and act on their expertise (Note: professional not organizational). |
|  | Accountability | The obligation or willingness of service providers to accept responsibility for their decisions and behaviours. |
|  | Adherence to Code of Ethics | The morals, principles, and values that an individual adheres to, which may define their interactions with others, and may be influenced by both personal and published professional codes of ethics. |
|  | Privacy and Confidentiality Obligation | A service provider’s obligation to respect privacy and commitment to maintaining the discretion of patient health information, whether written, verbal, electronic, photographic, or stored on any other medium. |
|  | Compulsion to Act | The need to “do something” whether or not effective care is available. |
|  | Job Satisfaction | Contentment and well-being in the workplace that exists among service providers. |
|  | Attitudes | Feelings, beliefs and viewpoints towards a particular person, situation, innovation, program, or change, often resulting from their beliefs and reflected in their behaviour. |
|  | Tolerance of Ambiguity | Comfort with ambiguous situations or uncertainty. |
|  | Organizational Commitment | An individual’s psychological attachment to the organization. |
|  | Buy-In to Change | Agreeing with and accepting a suggestion or change. |
|  | Personal Perception of Liability | Perceived personal threat of litigation or risk of a formal complaint being filed^2^. |
|  | Economic Incentive | e.g., fee-for-service for physicians |

**Table 2: Features of Context in ICON (Meso Level)**

| **Attribute** | **Example Features** | **Feature Description** |
| --- | --- | --- |
| **Domain: Organizational Climate and Structures** | | |
| Economic Arrangements | Funding Model | Consists of a financing mechanism; a paid workforce; information on which to base decisions and policies; facilities; and logistics to deliver quality medicines and technologies. |
|  | Costs of Healthcare Delivery | Expenditures or financial considerations (e.g., budgeting) taking the form of expenses. Costs are measured in money, but supply costs and labour expenditures are often related to or represented by objects and staff roles. |
|  | Funding/Endowment | To allocate or provide funds or endowment (e.g., in-kind financial support) for a program, project, or innovation, including the allocation of grants and/or government support. |
| Elements of Organizations | Type of Ownership | The type of proprietary authority or control body responsible for an organization. Considerations include if the organization is public/state vs. private, for-profit vs. not- for-profit, faith-based vs. not faith-based etc. The type of ownership can dictate how an organization is funded and managed, and may influence the organization's mission, values, goals, culture, and services offered (e.g., a religious-owned Catholic hospital may not offer abortion services). |
|  | Location | The location and situation of an organization. This can refer to the country (or region, province, state, etc.), where the organization is located (including multiple sites), and the characteristics of the locale(s) (e.g., rural, urban, etc.). |
|  | Organizational Size | The relative proportion or size of a given unit/facility/organization, often measured by number of staff, number of patients/clients/consumers served, number of beds, and/or number of units. |
|  | Setting/Type of Environment | The setting where service providers operate. This can include private clinics, hospitals, nursing care homes, public health practices, client homes (e.g., home care) and specialty practices (e.g., a Heart Institute). |
|  | Physical Structures | The tangible, physical infrastructure and facilities required for the operation of an organization (e.g., buildings, parking lots, power infrastructure, water supply, communications structures such as mainframes and servers, etc.). |
|  | Programs (Differentiation) | How care is organized in hospitals (e.g., cardiovascular, neurology). This includes how large numbers of people are clustered into smaller groups and differentiated, and how the independent actions of these differentiated groups are coordinated to produce a holistic product or service^3^. |
| Organizational Climate | Climate (Atmosphere) | The encompassing tone or mood of a place or situation^4^ in an organization as influenced by environmental cues (e.g., lighting, noise level), the design of space, and social climate (e.g., tension, calm demeanors), affecting the experience of the patient/client/consumer or service provider. |
|  | Team Climate | A supportive, cohesive atmosphere at work in which there is a shared sense of purpose, cooperation, and willingness to contribute to the common good^5,6^. |
|  | Conflict | An environment or situation where individuals are at variance with one another or disagree. |
| Physical and Technological Resources | Space | The presence/absence, design, maintenance, and allocation of areas for the provision of care in an organization. |
|  | Equipment &Supplies | Available resources (equipment and supplies) of whatever kind needed for the provision of services. |
|  | Technology | Systems, software, digital infrastructure and devices (and their interconnectedness) being applied for the purpose of providing services. |
|  | Online Resources | Readily accessible websites, databases, and/or other information sources available to a clinician or patient by means of the internet or other electronic network. |
|  | Evidence-based Resources | Existence of guidelines and other evidence-based guidance tools. |
|  | Documentation | Digital or printed documents used by team members to record, for example, treatments, tests, patient interactions, observations, and care plans. |
|  | Reminders | Memory aids and other environmental cues intended to facilitate the change or act as a cue for a particular behaviour or set of behaviours. |
| **Domain: Organizational Social Behaviour** | | |
| Internal Relationships | Social Networks | Includes the linkages and connections among organizations and other stakeholders that enables social support and the sharing of information and services among individuals and groups. |
|  | Social Capital | The sum of the resources, actual or virtual, that accrue to an individual or a group by virtue of possessing a durable network of more or less institutionalized relationships of mutual acquaintance and recognition^7^. |
|  | Partnerships (or Collaborative Practices) | A collaborative relationship between two or more parties based on trust, equality, and mutual understanding for the achievement of a specified goal^8,9^ (e.g., between service provider groups, and between staff and management). |
| Organizational Culture | Cultural Norms | The values and beliefs about the extent to which other people in the organization should or should not perform particular behaviours. |
|  | Shared Expectations | Common understanding between individuals. Rules that guide behaviour within social groups. |
| **Domain: Organizational Response to Change** | | |
| Organizational Change Processes | Formal Change Systems and Processes | The systems by which an organization or institution organizes and maintains the procedures and operations required to bring about change, including adequate process to obtain agreement on necessary implementation/ innovation decisions at all administrative, clinical, and operational levels of the organization^5^. |
|  | Quality Improvement Processes | Structured processes for evaluating the performance of systems and then determining needed improvements in both functional and operational areas^10^. Successful processes rely on the routine collection and analysis of data. |
|  | Engagement | The meaningful involvement of staff or stakeholders in the delivery of services and in change processes. |
|  | Champions/Opinion Leaders | One or more respected service provider(s) who actively advocates for the change and is involved in the change^11^. |
| Receptivity to Change | Change Culture | The extent to which an organization is "change friendly", including whether or not changes like the one in question are normally welcomed or met with cynicism and whether or not personnel are used to and comfortable with change^12^. |
|  | Tension for Change | The degree to which stakeholders perceive the current situation as intolerable or needing change^3^. |
|  | External Pressure for Change | Pressures perceived to come from external sources such as regulatory and funding^1^ or catastrophic events. |
|  | Readiness for Change | Shared resolve to implement a change (change commitment) and shared belief in their collective capability to do so (change efficacy)^13^. |
|  | Compatibility | The degree of tangible fit between meaning and values attached to an intervention by involved individuals, how those align with individuals' own norms, values, and perceived risks and needs, and how the intervention fits with existing workflows and systems^3^. |
|  | Change Saturation | When the number of changes being implemented exceeds the capacity of the individuals in an organization to effectively adopt and use those changes. |
| **Domain: Organizational Processes** | | |
| Communication Processes | Formal Communication | The exchange of information through prescribed, official, or predetermined means. Formal communication is governed by the established chain of command and takes place at pre-established times and places, examples: during rounds, at handover, newsletters. |
|  | Informal Communication (or Social Interactions) | The exchange of information not through prescribed, official, or predetermined means; examples: informal conversations^14^. |
|  | Social Influence | Processes by which individuals are affected by others’ social construction of events, ideas, objects, and behaviours and are subject to pressure to conform their behaviour, attitudes, and beliefs to that social reality^15^. |
|  | Advocacy | The act or process of supporting a cause or proposal^16^. |
| Evaluation Activity | Quality Improvement Monitoring | Quality improvement monitoring that includes job management, performance and integrity criteria, and identification of records and competencies such as staff knowledge, skills, experience, and qualifications. |
|  | Performance Measurement | The routine use of internal performance measures, audit, feedback, peer review, and/or external sources to measure performance at the individual, team, organizational, and system levels^17,18^. |
|  | Performance Feedback | Helpful information or criticism that is given to an individual or group to indicate when tasks have been done well and/or what can be done to improve a performance, product, etc. |
|  | Review of Employee Performance (Staff/Manager) | A staff performance review process is in place that enables staff reflection on practice and goal setting is regularly reviewed^11^ (e.g., managers review of staff and staff feedback on management). |
|  | Patient/Client/Consumer Feedback to Staff | Evaluative comments or feedback to service providers made by patients/clients/consumers and their families. This can include formal surveys, questionnaires, interviews, and informal conversations. |
| Governance | Organizational  Mission, Goals & Priorities | The statements, objectives, and values that guide an organization (e.g., professionalism, collaboration, mutual respect and diversity), define its purpose, determine what merits attention, outline expected outcomes, and often determine the allocation of resources. |
|  | Organizational Authority Structure | The typically hierarchical arrangement of lines of authority, communications, rights and duties of an organization. Organizational structure determines how the roles, power and responsibilities are assigned, controlled, and coordinated, and how information flows between the different levels of management^19^. |
|  | Power | The capacity or ability to direct or influence the behaviour of others^20^. |
|  | Standard of Practice or Care | The level of care that can be reasonably expected from a skilled provider in a particular situation. Standards of practice or care establish the minimum benchmark to which service providers should adhere in order to avoid charges of negligence^21^. |
|  | Internal Policies | A course of action, often in the form of documented and available protocols and procedures, that is proposed, developed, and adopted by an organization. |
|  | Incentives & Disincentives | Relates to incentives or disincentives embedded in regulatory policies, funding and reimbursement programs, and organization rules and policies themselves that alter the costs and benefits supporting new behaviours and practices. Incentives may be monetary or come in non-financial forms^22^. |
| Leadership | Leadership Styles | The style of leadership adopted by an individual (e.g., transformational, active, autocratic, etc.). |
|  | Formal Leaders | Has responsibility and authority for making the change. Does this person have the time, credibility and the technical-skills, people-skills and political skills to plan and carry through the change and adapt to surrounding changes?^11^ |
|  | Senior Leaders | A group of individuals within an organization who are in a high-level position of authority and who are expected to direct and control the overall functions of the organization. |
|  | Role Models | Any behaviour performed by a service provider and motivated by the desire to set an example, or embody best practices, for a student, a junior, or a new team member. |
|  | Mentors | A relationship established between a leader or superior and a subordinate or trainee, characterized by a close, and typically enduring, pedagogical exchange, where the subordinate or trainee learns by observing and regularly communicating with the leader or superior. |
| Management | Formal Planning | Formal planning in the organization relating to the use of the innovation (e.g., in policy plans, work plans, strategic plans, annual goals, etc.)^23^. |
|  | Management Support | Assistance, guidance, direction, encouragement, and/or validation (physical, psychosocial, emotional, etc.) provided to staff by management. |
|  | Use of Resources | Efficient, excessive or under use of resources. |
| Organization of Work | Workload | The amount of work to be performed by a service provider team. |
|  | Adequacy of Staff Composition | The availability of a sufficient number of providers with the necessary skills to provide appropriate care. |
|  | Support Personnel | Readily accessible personnel in the workplace that support achieving the organization’s mission (e.g., Environmental Services, Food Services, Human Resources, Educators, Expert/Consultant, etc.). |
|  | Teamwork | The extent to which service providers of varying roles and skill sets work together to achieve a common aim. |
|  | Scheduling | Designated work times, the arrangement of work times among a service provider team (including shift work), and other on-call arrangements. |
|  | Workflow | The sequence of industrial, administrative, or other processes through which a piece of work passes from initiation to completion^24^. |
|  | Work Tempo | The characteristic rate, rhythm, unpredictability or pattern of activity among a service provider team; the pace at which work is carried out. |
|  | Time | Time required for the completion of work tasks, and as it is managed by staffing and the arrangement of work. |
| System Processes | Quality Assurance Process | System of maintaining standards in products and services and monitoring of related activity in a structured and strategic manner such that problems can be identified and resolved before long-term issues are realized. |
|  | Project Management | The practice of initiating, planning, executing, controlling, and closing the work of a team to achieve specific goals and meet specific success criteria at the specified time^25^. |
|  | Optimizing Standardization of Care | Efforts to coordinate and systematize the work practices, information, and care procedures required for the delivery of services. |
|  | Continuity of Care | Continuity of care is the degree to which a series of events is experienced as coherent and connected, and consistent with the patient/client/consumer’s needs and personal context^26^. |
|  | Organizational Training and Education | Investment in organized activity provided by an institution for its employees aimed at imparting information and/or instructions to improve the recipient's performance or to help them attain a desired level of knowledge or skill (e.g., training about a new innovation). |
|  | Process Complexity | Includes process length (the process contains sequential sub-processes), process breadth (the number of choices presented at decision points in the process), and delivery systems^27^. |
|  | System Complexity | Includes the number of potential organizational units (teams, clinics, departments) or person types (providers, patients, managers) that may be the foci for interventions^27^ as well as the interrelatedness of the components of a system, and relationships between a system's parts give rise to its collective behaviours and how the system interacts and forms relationships with its environment. |

**Table 3: Features of Context in ICON (Macro Level)**

| **Attribute** | **Example Features** | **Feature Description** |
| --- | --- | --- |
| **Domain: External Influences** | | |
| Community Influences | Public Influences | The general level of social knowledge and attitude as it regards to a particular clinical behaviour or procedure (e.g., public attitudes towards the healthcare system or about organ donation, or a public reaction to a hospital audit as it has been portrayed in the media). |
|  | Peer Organizational Pressure | Competitive pressure to implement an intervention from any outside entity with which the organization feels some degree of affinity or competition at some level within their organization^3^. |
| Intercommunity/  Interorganizational/  Intersectoral Relationships | Intersectoral Collaboration | The partnerships and collective actions by individuals from health and other government sectors, private, voluntary, and non-profit groups within the same or different factions or constituencies to improve health of populations. May be referred to as coalitions, cooperative initiatives, alliances, or partnership^28,29^. |
|  | Community Health Outreach | The collaboration between individuals or an organization with a community to enact a temporary and mobile project that is intended to increase access to services to individuals or groups who face unequal or unequitable access to care than the general patient/client/consumer population^30^. |
|  | Coordinated Action | The extent by which differing organizations communicate and tailor their interventions to meet mutual needs^31^ or achieve a synergistic effect^32^. |
| Political Influences | Politics | The governance and public affairs landscape of a given region or community, including the local political party in power and the individual political personality types of those in power, as well as interactions between those in power and those competing for power. |
|  | Political Climate | The aggregate, current mood and opinions of a populace about political issues that also currently affect that population, in a general sense. |
|  | Complexity of the Broader  Sociopolitical Environment | The interrelatedness and intricacy of the components of the sociopolitical system. These elements exist and interact both within and outside organizations. |
| Regulatory Influences | Laws & Legislation | Established legal statutes outlining the prerogatives and responsibilities of service providers, organizations, and the rights of patients/clients/consumers (within organizations). |
|  | External Policies, Directives, Mandates, and Regulations | Statutes or principles established and enforced by an agency external to the organization. Policies, directives, mandates, and regulations can be enforced at the local, state/provincial, national, or international level and may or may not be governmental. These standards are often binding, sometimes based on law or remuneration structures, and are outside the control of organizations. |
|  | Industrial Influences | E.g., unionization, collective bargaining. |
|  | Accreditation Standards | The predetermined criteria and standards that an organization must meet in order to be certified to deliver services. |
| Regional/Global Influences on Health | Epidemics/Pandemics/  Endemics/Outbreaks | Health emergencies at a population level which affect large groups of people and thus have impacts on organizations. Epidemic (affects a number of people within a community, population, or region). Pandemic (epidemic that’s spread over multiple countries or continents). Endemic (restricted to a particular group or country). Outbreak (greater-than-anticipated increase in the number of endemic cases). |
|  | Natural Disasters | A natural event such as a flood, earthquake, or hurricane that causes great damage or loss of life. |
|  | Climate Change | A change in global or regional climate patterns; attributed largely to increased levels of atmospheric carbon dioxide produced by the use of fossil fuels. |

**References**

1. Simpson DD, Dansereau DF. Assessing organizational functioning as a step toward innovation. *Sci pract perspect.* 2007;3(2):20.

2. Oxman A, Flottorp S, . An overview of strategies to promote implementation of evidence-based health care. In: Silagy C, Haines A, eds. *Evidence-based Practice in Primary Care.* 2 ed.: BMJ Books; 2001:101-119.

3. Damschroder LJ, Aron DC, Keith RE, Kirsh SR, Alexander JA, Lowery JC. Fostering implementation of health services research findings into practice. A consolidated framework for advancing implementation science. *Implement Sci.* 2009;4:50.

4. Cambridge Dictionary. Atmosphere. 2022; <https://dictionary.cambridge.org/dictionary/english/atmosphere>. Accessed September 29, 2022, 2022.

5. Solberg LI, Brekke ML, Fazio CJ, et al. Lessons from experienced guideline implementers: attend to many factors and use multiple strategies. *Jt Comm J Qual Patient Saf.* 2000;26(4):171-188.

6. Wallin L, Estabrooks CA, Midodzi WK, Cummings GG. Development and validation of a derived measure of research utilization by nurses. *Nurs Res.* 2006;55(3):149-160.

7. Bourdieu P, Wacquant LJ. *An invitation to reflexive sociology.* University of Chicago press; 1992.

8. Episcopal Church Foundation. Partnerships. n.d.; <https://www.ecf.org/partnerships#:~:text=Partnerships%20ECF%20believes%20a%20collaborative%20relationship%20between%20two,as%20well%20as%20benefits%2C%20making%20shared%20accountability%20critical>. Accessed September 29,2022, 2022.

9. Cochrane Community. Partnerships policy. 2016; <https://community.cochrane.org/organizational-info/resources/policies/organizational-position-statements/partnerships-policy>. Accessed September 29, 2022, 2022.

10. Eby K. Quality Improvement Processes: The Basics and Beyond. 2021; <https://www.smartsheet.com/quality-improvement-process>. Accessed September 29, 2022, 2022.

11. Ovretveit J. Change achievement success indicators (CASI). In: Stockholm, Sweden: Karolinska Institute Medical Management; 2004.

12. Ovretveit JC, Shekelle PG, Dy SM, et al. How does context affect interventions to improve patient safety? An assessment of evidence from studies of five patient safety practices and proposals for research. *BMJ Qual Saf.* 2011;20(7):604-610.

13. Weiner BJ. A theory of organizational readiness for change. *Implement Sci.* 2009;4(1):67.

14. Estabrooks CA, Squires JE, Cummings GG, Birdsell JM, Norton PG. Development and assessment of the Alberta Context Tool. *BMC Health Serv Res.* 2009;9:234.

15. Aarons GA. Measuring provider attitudes toward evidence-based practice: consideration of organizational context and individual differences. *Child Adolesc Psychiatr Clin N Am.* 2005;14(2):255-271, viii.

16. Merriam Webster Dictionary. Advocacy. 2020. Accessed June 2020, 2020.

17. Kitson AL, Rycroft-Malone J, Harvey G, McCormack B, Seers K, Titchen A. Evaluating the successful implementation of evidence into practice using the PARiHS framework: Theoretical and practical challenges. *Implement Sci.* 2008;3(1).

18. Stetler CB, Damschroder LJ, Helfrich CD, Hagedorn HJ. A Guide for applying a revised version of the PARIHS framework for implementation. *Implement Sci.* 2011;6:99.

19. Business Dictionary. Organizational Structure. 2022; <https://businessdictionary.info/definition/organizational-structure/>. Accessed September 29, 2022, 2022.

20. Dictionary.com. Power. 2022; <https://www.dictionary.com/browse/power>. Accessed September 29, 2022, 2022.

21. Vanderpool D. The Standard of Care. *Innov Clin Neurosci.* 2021;18(7-9):50-51.

22. Mendel P, Meredith LS, Schoenbaum M, Sherbourne CD, Wells KB. Interventions in organizational and community context: A framework for building evidence on dissemination and implementation in health services research. *Adm Policy Ment Health.* 2008;35(1-2):21-37.

23. Fleuren MA, Paulussen TG, Van Dommelen P, Van Buuren S. Towards a measurement instrument for determinants of innovations. *Int J Qual Health Care.* 2014;26(5):501-510.

24. Oxford English Dictionary. Workflow. n.d.; <https://www-oed-com.proxy.bib.uottawa.ca/view/Entry/400203?redirectedFrom=workflow#eid>. Accessed July 18, 2022.

25. Tilsed M. Project management. 2016; <https://www.linkedin.com/pulse/project-management-discipline-initiating-planning-malcolm?articleId=6159452121843458048>. Accessed September 29, 2022, 2022.

26. Health Quality Council of Alberta. Continuity of Patient Care. 2020. Accessed June 8 2020, 2020.

27. Kochevar LK, Yano EM. Understanding Health Care Organization Needs and Context: Beyond Performance Gaps. *J Gen Intern Med.* 2006;21(Suppl 2):S25-S29.

28. Kramer JS, Philliber S, Brindis CD, et al. Coalition models: Lessons learned from the CDC’s community coalition partnership programs for the prevention of teen pregnancy. *J Adolesc Health.* 2005;37(3):S20-S30.

29. Public Health Agency of Canada. Key Element 6: Collaborate Across Sectors and Levels. 2016. Accessed March 8, 2021, 2021.

30. Shin HY, Kim KY, Kang P. Concept analysis of community health outreach. *BMC Health Serv Res.* 2020;20(1):1-9.

31. Marsick VJ, Watkins KE. Demonstrating the value of an organization's learning culture: the dimensions of the learning organization questionnaire. *Adv Dev Hum Resour.* 2003;5(2):132-151.

32. Brown EC, Hawkins JD, Arthur MW, Abbott RD, Van Horn ML. Multilevel analysis of a measure of community prevention collaboration. *Am J Community Psychol.* 2008;41(1):115-126.
