# Supplementary material for: The Implementation in Context (ICON) Framework: A meta-framework of context domains, attributes and features in healthcare
Source: Health Res Policy Syst. 2023 Aug 7;21:81. doi: 10.1186/s12961-023-01028-z (PMC10408185; doi:10.1186/s12961-023-01028-z)
Supplement: Supplementary file 5 — Additional file 5. ICON Qualitative Screening Tool. [file 12961_2023_1028_MOESM5_ESM.docx]

**Additional File 5**

**ICON Qualitative Screening Tool**

This tool is designed to assist individuals gain a broad overall understanding of the aspects (attributes and features) of their context that they may want to prioritize for detailed quantitative investigation prior to implementing a specific implementation intervention. The tool can be self-administered to increase one’s own self-awareness of their context or can be administered to key informants in an organization in interview or focus format. All screening questions should be asked in relation to the specific implementation intervention being considered.

The context attributes being addressed are in brackets after the question. Additional prompts are available in the ICON Framework in the form of additional context features, please see the full listing of context features corresponding to each attribute in ICON. Definitions of all attributes and features are available in the ICON framework (Table 1-3 of the manuscript and Additional file 1).

Notes:

- This tool is not meant to provide an in-depth assessment of one’s context. This is a screening tool and as such is meant to provide an overview of context that enables its users’ to more easily prioritize the attributes of context that may require more detailed assessments.
- This screening tool has been pretested by 10 knowledge users in Canada (who self-administered the tool and who also administered it to staff in their organizations in interview format). The tool took, on average, 20 minutes to complete in interview format. Comments received from users on the tool were positive; that it increased their awareness of some of the areas in their context that they did not realize were issues and thus, need to pay closer attention to for implementation of the intervention they were considering.

**Questions**

1. What are the characteristics of the patients in this clinical setting that may influence whether, how and with what supports you would be able to implement [the specific KT initiative]?

*[prompt with the following as appropriate]*

- 1. **Patient demographics** (e.g., ethno-cultural profile) *[Attribute: Patient/Client/*

*Consumer Population]*

- 1. **Patients’ conditions and functioning** (e.g., condition severity and average/range of functioning)

*[Attribute: Patient/Client/Consumer Population]*

- 1. **Patients’ beliefs** (e.g., medical model) and values (e.g., patient-centered care) *[Attribute: Patient/Client/Consumer Population]*

1. What are the characteristics of the health professionals in this clinical setting that may influence whether, how and with what supports you would be able to implement [the specific KT initiative]?

*[prompt with the following as appropriate]*

- 1. **Professionals’ beliefs and values** *[Attribute: Service Provider Population]*
  2. **Professionals’ knowledge and skills profile** *[Attribute: Service Provider Population]*

1. What are the characteristics of your organization that may influence whether, how and with what supports you would be able to implement [the specific KT initiative]?

*[prompt with the following as appropriate]*

Organizational factors

- 1. **Organizational demographics** (e.g., number of sites) *[Attribute: Elements of Organizations]*
  2. **Organizational behaviours** (e.g., cohorting patients) *[Attribute: Organizational Culture]*
  3. **Leadership, management and coordination**, **type of leadership** (e.g., levels of management, responsibilities for management (e.g., logistics support) *[Attributes: Leadership, Management]*
  4. Organizational **culture**, including organizational norms and values about using evidence, etc. *[Attribute: Organizational Culture]*
  5. Organization’s **financial arrangements** (e.g., funding streams, professional remuneration, costs) *[Attribute: Economic Arrangements]*
  6. Organization’s **governance arrangements** (i.e., who makes what types of decisions in the organization, including clinical and corporate governance) *[Attribute: Governance]*
  7. Organization’s **social infrastructure** (e.g., standardization of care, continuity of care, organizational mission, goals and priorities) *[Attributes: Organizational Culture*, *System Processes, Governance]*
  8. Organization’s **support networks** (e.g., organizational training and education programs, support from management) *[Attribute: System Processes, Management, Internal Relationships]*

1. Thinking about how you might approach implementing [the specific KT initiative], what would be the characteristics of what you would do, and when and how you would do it that may influence whether, how and with what supports you could implement [the specific KT initiative]?

*[prompt with the following as appropriate]*

- 1. **Programs and services** (e.g., soup to nuts vs. focused factory) that you provide *[Attribute: Service Provider Population]*
  2. **Work environment** characteristics (e.g., scheduling and organizational training and education) *[Attributes: Organizational Culture, Organization of Work, System Processes]*
  3. **Resource-management** characteristics (e.g., slack resources) *[Attribute: Physical and Technological Resources]*
  4. **Information-sharing** characteristics (e.g., at shift change) *[Attribute: Communication Processes]*
  5. **Physical infrastructure**, ranging from information resources, technology (e.g., electronic health records), equipment, and physical space *[Attribute: Physical and Technological Resources]*
  6. **Human resources,** including type (e.g., support staff) and number *[Attribute: Organization of Work]*

1. What characteristics associated with the complexity of your organization might influence whether, how and with what supports you could implement [the specific KT initiative]? *[Attribute: System Processes]*

*[prompt with the following as appropriate]*

- 1. **Organizational politics** *[Attribute: Governance]*
  2. **Evaluation** landscape within the organization (e.g., quality measurement processes, other pressures for change, receptivity to change, and quality improvement / change management processes) *[Attributes:* *Evaluation Activity,* *Receptivity to Change,* *Organizational Change Processes]*

1. What characteristics of the broader health or political system external to your organization might influence whether, how and with what supports you could implement [the specific KT initiative]? *[Attributes: Evaluation, Politics & Power; Market; Complex System; Collaborative Relationships]*

*[prompt with the following as appropriate]*

Placeholder for things like:

- 1. System-level **governance arrangements** (e.g., nurses’ regulated scope of practice) *[Attribute: Governance]*
  2. **Political** factors (e.g., political interference in organizational decisions) *[Attribute: Politics & Power]*

1. Are there any other contextual factors that might influence whether, how, or with what supports you could implement [the specific KT initiative]?
